# Development and testing of a novel image analysis algorithm for descriptive evaluation of shape change of a shrinkable soft material

**DOI:** 10.1038/s41598-021-97141-6

**Published:** 2021-09-13

**Authors:** Pinpinat Stienkijumpai, Maturada Jinorose, Sakamon Devahastin

**Affiliations:** 1 Department of Food Engineering, School of Engineering, King Mongkut’s Institute of Technology Ladkrabang, 1 Soi Chalong Krung 1, Ladkrabang, Bangkok, 10520 Thailand; 2 Advanced Food Processing Research Laboratory, Department of Food Engineering, Faculty of Engineering, King Mongkut’s University of Technology Thonburi, 126 Pracha u-tid Road, Tungkru, Bangkok, 10140 Thailand; 3 The Academy of Science, The Royal Society of Thailand, Dusit, Bangkok, 10300 Thailand

**Keywords:** Chemical engineering, Characterization and analytical techniques

## Abstract

Soft material can undergo non-uniform deformation or change of shape upon processing. Identifying shape and its change is nevertheless not straightforward. In this study, novel image-based algorithm that can be used to identify shapes of input images and at the same time classify non-uniform deformation into various patterns, i.e., swelling/shrinkage, horizontal and vertical elongations/contractions as well as convexity and concavity, is proposed. The algorithm was first tested with computer-generated images and later applied to agar cubes, which were used as model shrinkable soft material, undergoing drying at different temperatures. Shape parameters and shape-parameter based algorithm as well as convolutional neural networks (CNNs) either incorrectly identified some complicated shapes or could only identify the point where non-uniform deformation started to take place; CNNs lacked ability to describe non-uniform deformation evolution. Shape identification accuracy of the newly developed algorithm against computer-generated images was 65.88%, while those of the other tested algorithms ranged from 34.76 to 97.88%. However, when being applied to the deformation of agar cubes, the developed algorithm performed superiorly to the others. The proposed algorithm could both identify the shapes and describe their changes. The interpretation agreed well with that via visual observation.

## Introduction

Shape is among the most important characteristics that must be carefully designed and controlled when manufacturing a product, especially when a shrinkable soft material is used as a starting raw material^1^. This is because such a material consists mainly of water, which may need to be removed (or, in other words, dehydrated) during processing. Material structure, e.g., cell wall of plants or even soft structure of various gels, which holds and is indeed supported by water would then collapse, resulting in shrinkage and deformation^2–6^. Since moisture gradients generally exist during dehydration due to different rates of moisture removal at different locations, deformation is in most cases non-uniform^2,7^. This leads in turn to shape change of a resulting product, which may be either desirable or undesirable, depending on a specific manufacturing situation and purpose.

Non-uniform deformation is of interest as such a deformation would lead to changes in both size and shape of a product undergoing processing. These changes in turn affect heat and mass transfer behavior as well as many other properties of a product^8–10^. A means to accurately characterize and describe this type of deformation is therefore highly desirable. Deformation is nevertheless typically determined and reported in terms of volumetric shrinkage, which is a ratio of volume change of a specimen to its initial volume^11,12^. However, volumetric shrinkage cannot clearly be used to quantify the change of shape (i.e., non-uniform deformation). It has indeed been reported that the same specimen undergone two different processes and/or conditions could have the same volumetric shrinkage but completely different shapes^11,13^.

Image analysis is an alternative method that can be used for material and product characterization; the method can well be used for the determination of both size and shape of a product^14,15^. Nevertheless, although there exist several algorithms that can be used to identify shape, e.g., template matching method^14^, or such statistical methods as k-cluster and discriminant analyses^14,16^ as well as decision tree^14,17–19^, such available algorithms can only classify images into a limited number of predetermined shapes, making these algorithms less robust and flexible. The limitation exists because in order to evaluate shape using image analysis, appropriate shape parameters must first be devised and tested. Although various shape parameters have been proposed^9,14,17^ most reported works were performed using shape parameters that can only describe or identify some specific shapes. In other words, each parameter has its own set of shapes that can be identified; therefore shape must be indicated a priori^20^, which is clearly an inappropriate approach. Most available algorithms cannot also be used to describe non-uniform deformation; only a period over which non-uniform deformation takes place could be identified. In addition, in a real-world situation, a product entering an image analysis system may be arbitrarily oriented^17,18,21^; this may lead to an inaccurate decision during the shape matching process. Therefore, an input image needs to be properly aligned prior to being analyzed for more accurate results^22^. Most existing algorithms lack this important image processing step, however.

More robust neural network-based algorithms such as convolutional neural networks (CNNs) have recently been used to identify shapes of input images. CNNs have successfully been used, for example, to identify shapes of crystal structures^23,24^. Performance of CNNs nevertheless depends on their architectures; various architectures, including GoogLeNet, ResNet-50, Xception, VggNet-19, AlexNet and EfficientNet-16 have been studied^25,26^. More importantly, for CNNs to succeed in real-world situations, a large number of images are required to train the networks. Such a training (and subsequent validation) step may require large computational resources and time, hence preventing the use of CNNs in a situation where limited developmental time and resources are available. Acquiring a sufficient number of real-world images may also sometimes not be feasible; this may in turn adversely affect the identification accuracy^27^. More importantly, most available algorithms cannot be used to describe the evolution of non-uniform deformation; only the shape (and not its evolution) can be described and quantified.

The present study aimed at developing a novel image-based algorithm that can be used to determine the shapes of input images and at the same time capture changes in the shapes (non-uniform deformation) of such images. The effects of alignment and alignment methods on the resulting shape analysis results were investigated. Newly proposed scheme that can be used to classify non-uniform deformation into various patterns, i.e., swelling/shrinkage, horizontal and vertical elongations/contractions as well as convexity and concavity, is also proposed. The developed algorithm was first tested with computer-generated images and later applied to cubes of agar gel of different compositions, which were used as a model highly shrinkable soft material, undergoing drying at different temperatures. Analysis results were compared with those obtained using shape parameters and shape-parameter based algorithm as well as CNNs.

## Results and discussion

### Test of shape identification algorithms

First, the accuracies of the newly proposed shape identification procedures with and without image alignment were compared; the results are shown in Table 1. The results of the use of the algorithm by Igathinathane et al.^17^ and CNNs are also listed. Note that the shape identification accuracy was calculated by comparing the known shapes of the computer-generated images with those identified by each tested algorithm. A count was made on both correct and incorrect shape identifications; percent accuracy was then calculated. In the case of the newly developed algorithm, identifying the shape of input images without any alignment yielded the lowest accuracy, as expected. Unaligned images had higher chances of being overlapped with other reference geometries than with the most suitable ones. Nevertheless, although the alignment step could help improve the accuracy of shape identification, such a step resulted in a longer processing time. Zigzagging artefact, which was the results of the alignment step^28^, might also affect shape identification.

**Table 1:** Accuracies of various shape identification algorithms.

Deformation pattern	Number of images		% Accurate identification						
			Without alignment	With alignment			Igathinathane et al.^12^	CNN	
				EEM	Extrema	Manual		ResNet-50	Xception
0, 0, 0, 0, 0	120		35.00	47.50	100.00	86.67	40.00	100.00	100.00
+, +, +, 0, 0	240		32.50	46.67	85.00	83.33	40.00	100.00	100.00
+, +, 0, 0, 1	240		32.08	40.00	70.00	98.33	40.00	100.00	100.00
+, 0, +, 0, 1	240		32.50	59.58	82.92	100.00	40.00	100.00	100.00
0, 0, 0, −, 1	240		30.42	44.17	92.92	74.17	41.67	100.00	100.00
+, +, +, −, 1	480		35.21	45.00	85.83	82.08	41.04	100.00	100.00
+, +, 0, −, 1	480		33.96	44.17	68.13	87.50	35.83	100.00	100.00
+, 0, +, −, 1	480		32.92	60.00	80.83	86.88	26.46	99.79	100.00
0, 0, 0, +, 1	240		27.92	40.42	50.00	67.50	20.00	41.67	84.58
+, +, +, +, 1	480		26.04	39.58	48.54	60.63	20.00	100.00	100.00
+, +, 0, +, 1	480		25.83	28.96	42.71	61.42	40.00	52.08	92.50
+, 0, +, +, 1	480		30.63	56.88	35.00	62.71	40.00	50.21	96.67
	4200	Avg	30.98	45.93	65.88	77.10	34.76	85.48	97.88

EEM = equivalent ellipsis method.

**Figure 1:**
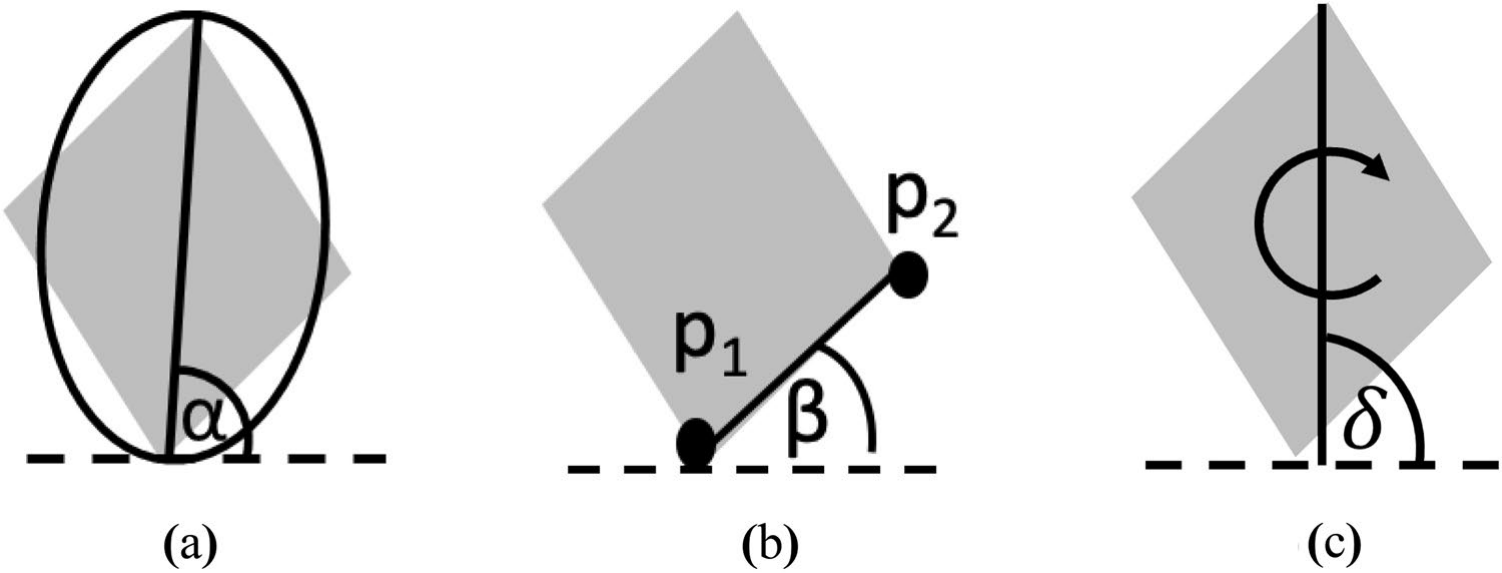
Image alignment methods**.** (**a**) Equivalent ellipsis method, (**b**) extrema method and (**c**) manual alignment method.

Different alignment methods (see Fig. 1) yielded different levels of accuracy as shown in Table 1. Equivalent ellipsis method resulted in the lowest accuracy. When images were not deformed (0, 0, 0, 0, 0), major axis of those images could have already been aligned, either on the horizontal or vertical axis, causing the method not to rotate the images until reaching α = 0° or even rotating the images upside-down; this is because α is always the acute angle between the major axis and horizontal axis. When applying the equivalent ellipsis method to images with horizontal elongation (+, +, 0, 0, 1), the method yielded the lowest accuracy. For example, when a triangle or pentagon was horizontally elongated, the method identified it instead as a rectangle or hexagon. Such an error occurred because the apex angle would change from smaller than 90° to larger than 90°. The method performed better when images were vertically elongated (+, 0, +, 0, 1) because the major axis of those images would align on the longest axis of the images. Nevertheless, this alignment method required the shortest processing time (around 0.25 s per image using the currently employed hardware) because it refers to only one angle (α) in order to perform the alignment process.

**Figure 2:**
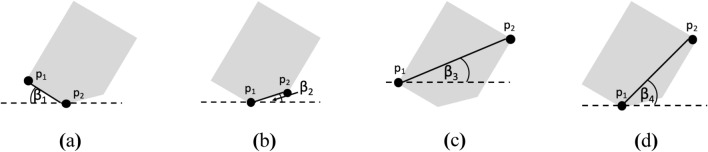
Possible β angles to refer to during alignment using extrema method.

Extrema method was noted to give more accurate identification results than the equivalent ellipsis method. The accuracy was higher than 50% if input images resembled their reference geometric shapes. This is because the extrema points of the images would be the same as those of the reference geometric images. On the other hand, when images were convexed (0, 0, 0, +, 1), the extrema points sometimes changed to the convex points. Convexity also caused images to look more like a circle or an ellipse. This alignment method required longer processing time than the equivalent ellipsis method (around threefold longer) as the former needs to refer to up to four angles to perform the alignment process (see Fig. 2).

Manual alignment method gave the highest accuracy (higher than 60% in all cases). The major advantage of this method arose when images experienced horizontal (+, +, 0, 0, 1) or vertical elongation (+, 0, +, 0, 1); such images would suffer less inaccuracy than the ones with no deformation (0, 0, 0, 0, 0). This is because the manual alignment method rotates an image from 0° to 360° at 1° interval, causing some zigzagging artefact on the image edge. However, when images were elongated, the dimension ratio of the artefact to the characteristic length of the images would decrease. Nevertheless, this method required the longest time, 51.29 s per image or around 70-fold longer than the extrema method. This is expected as it has 360 possible angles to refer to.

Although the manual alignment method resulted in the highest accuracy, the processing time was excessive. This is clearly not appropriate, especially if real-time image-based process control is to be conducted. For this reason, extrema method was chosen because it exhibited higher accuracy than the equivalent ellipsis method and yet performed 70 times faster than the manual alignment method.

It is seen in Table 1 that the developed algorithm (employing the extrema alignment method) gave 100.00% and 92.92% accuracy when input images did not suffer any deformation (0, 0, 0, 0, 0) and when images were concaved (0, 0, 0, −, 1), respectively. This is because the points at the angles of those images were distinct (except in the case of circles). This in turn caused the RMSD values to be lower. Swollen images (+,  , +, 0, 0) having more zigzagging edges, caused by the forced deformation process, would, for example, result in pentagons or hexagons looking more like circles. Horizontal elongation exhibited stronger adverse effect than vertical elongation because when images with apex angles of less than 90° (such as triangles and pentagons) were horizontally elongated, the angles would become larger than 90°. This in turn affected RMSD because this value was calculated from *d* values at the same angle θ. The algorithm did not well identify images when they were convexed as the image would become more like circles.

When all 4200 computer-generated images were analyzed using the developed algorithm with the extrema method, the overall capability to accurately identify the shapes of input images was noted to be 65.88%. In contrast, when the algorithm by Igathinathane et al.^17^ and CNN with ResNet-50 architecture (CNN ResNet-50) and CNN with Xception architecture (CNN Xception) were used for the same shape identification purpose, the overall accuracies were noted to be 34.76%, 85.48 and 97.88% respectively. The reason for the much lower overall accuracy of the algorithm by Igathinathane et al.^17^ is that the algorithm could not identify pentagons and hexagons as these shapes are not included in the algorithm; computer-generated pentagons and hexagons were therefore incorrectly identified as either ellipses or rectangles. This clearly indicates the disadvantage of this (or similar algorithms) where new reference geometric shapes always need to be added and analyzed when dealing with input images of peculiar shapes. In the case of CNNs, the algorithms were not good at identifying both horizontally and vertically convexed images. This may probably be due to the inherently ambiguous sorting classes and blurring appearance^29^. CNN Xception performed better than CNN ResNet-50, as expected^30^. It is important to note, however, that both the newly developed algorithm and that of Igathinathane et al.^17^ require no training, while CNN ResNet-50 and CNN Xception require significant training time. Using the currently employed hardware, as much as 41,942 and 138,269 s were required, respectively, for the two CNNs.

### Comparative performance of developed algorithm and existing shape parameters

#### Effect of size change

The effect of the change in image size on the Extent was first evaluated. Size change did not significantly affect the Extent. This is because when the size of an image increased, the size of the rectangular bounding box also increased at the same rate. Each geometric shape was noted to exhibit its own Extent value; the values for a triangle, rectangle, pentagon, hexagon and circle are 0.525 ± 0.003, 1.000 ± 0.000, 0.710 ± 0.002, 0.768 ± 0.002 and 0.805 ± 0.004, respectively. Extent nevertheless has a significant disadvantage that it can only be used to identify reference geometric shapes. This shape parameter cannot be used to describe non-uniform deformation; only a point where non-uniform deformation starts to take place could be identified^15^.

In the case of fractal dimension, change of this parameter could be divided into three periods based on the ratio of area of interest (AOI) to the total area of an image. When the aforementioned ratio was lower than 5%, fractal dimension was close to unity. This unexpected result was noted because the method that was used to calculate the fractal dimension here was the box counting method. When the size decreased, AOI resembled a point. This implies that fractal dimension calculated by the box counting method could not be used when the ratio of AOI to the total area of an image is lower than 5%. On the other hand, when such a ratio was higher than 5% but lower than 20%, fractal dimensions of a triangle, rectangle, pentagon, hexagon and circle were noted to be 1.826 ± 0.061, 1.863 ± 0.036, 1.852 ± 0.052, 1.869 ± 0.051, 1.883 ± 0.051, respectively. Finally, when such a ratio was higher than 20%, all fractal dimensions approached 2.

In the case of the presently developed algorithm, the change in size affected i, j and k. This is expected as i is related to image area, which is in turn related to size; j and k are also linearly related to horizontal and vertical dimension changes. Parameter l, which is used to indicate convexity and concavity was not affected by the change in size, as expected.

#### Effects of horizontal and vertical length changes

Both horizontal elongation and contraction did not affect the Extent because these changes equally affected both AOI and its rectangular bounding box. In the case of the newly developed algorithm, horizontal elongation and contraction affected i and j but did not affect k and l. When an image was horizontally elongated or contracted, its size also increased or decreased, respectively. However, since the change occurred only in the horizontal direction, k was not affected. Parameter l was also not affected as no convexity took place. The above-mentioned reasons can also be used to explain the effect of vertical length change.

#### Effects of convexity and concavity

Extent of a triangle was most significantly affected and so can be used to quantify both the convexity and concavity. In contrast, the Extent of a rectangle exhibited similar patterns of changes, whether convexity or concavity took place; Extent cannot therefore be used to classify convexity and concavity of this geometric shape. Extent values of a concaved pentagon, hexagon and circle exhibited similar patterns; however, the values belonging to these shapes that were made to convex remained almost unchanged. Therefore, the Extent can only be used to identify concavity of these shapes.

Since each geometric shape exhibited its own Extent value, convexity and concavity of a triangle could, for example, be identified by looking at the deviation of its Extent value from the reference value of 0.525; higher Extent value than the reference value indicated convexity and vice versa. Similar identifications can also be made for a pentagon, hexagon and circle. Since convexity and concavity caused the image perimeter to be rougher, fractal dimension increased. However, this parameter cannot be used to describe such a change of shape, only being able to identify if any change exists.

In the case of the presently developed algorithm, concavity and convexity only affected parameter l. As a result, this parameter can be used to identify and quantify convexity and concavity by looking at the deviation of its value from zero in much the same way as in the case of Extent. However, this parameter can be applied to all tested shapes, including a rectangle.

Note that parameter m is used to identify if deformation is uniform or non-uniform. Such a decision is made based on the values of α and l. Uniform deformation is said to take place only if both the value of α is higher than 95% and the value of l is higher than 99%. The chosen threshold of 95% is based on the typical confidence level used in most engineering statistical analysis. The threshold of 99% was, on the other hand, chosen by adjusting the value until the computed identification matched that visually observed.

#### Test of developed algorithm on agar cubes

**Figure 3:**
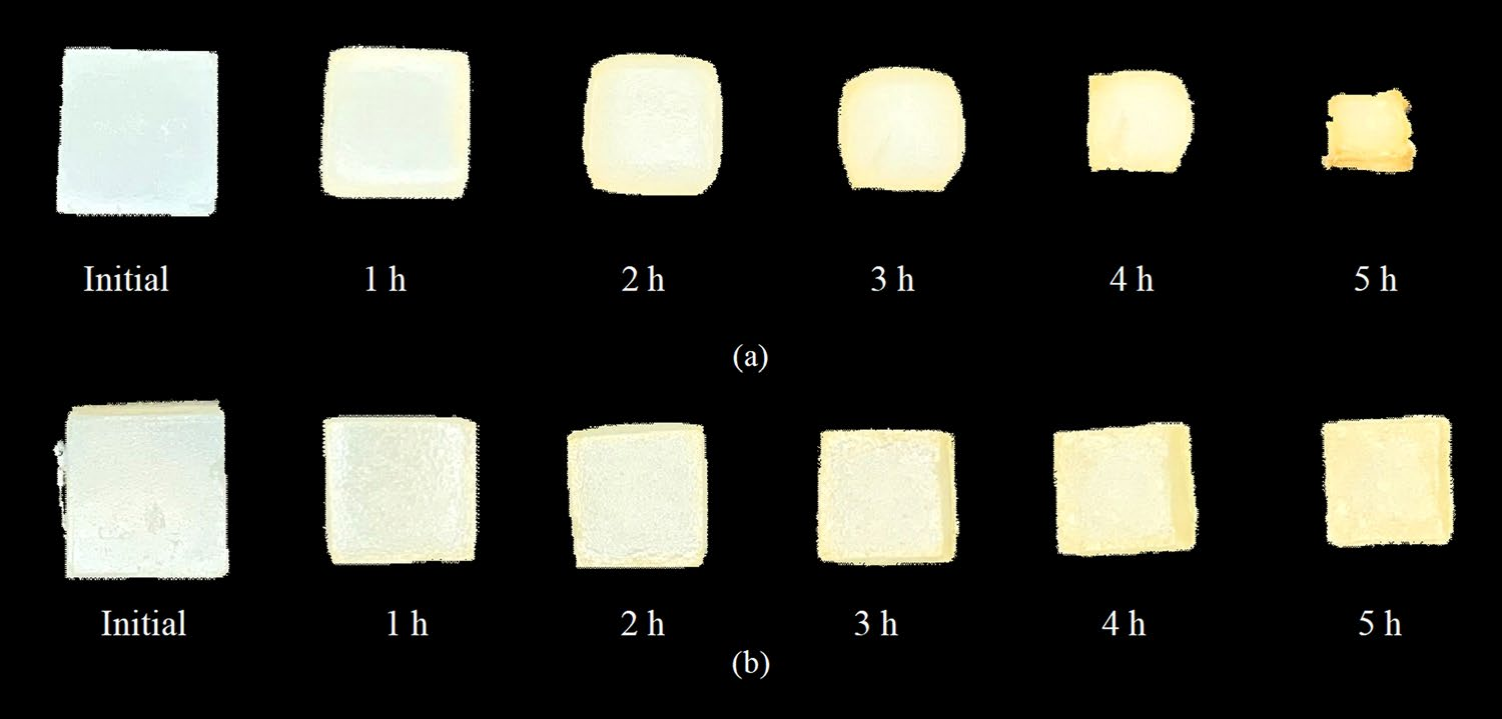
Deformation of agar cubes with (**a**) 0% sugar and (**b**) 20% sugar during drying at 80 °C.

Performance of the developed algorithm was evaluated by applying it to agar cubes containing either 0 or 20% sucrose undergoing hot air drying at 80 °C (see Fig. 3). Top-view images of agar cubes containing 0 and 20% sucrose are shown in Table 2. The algorithm indicated that the top-view shape of agar cubes with 0% sucrose changed from square to rectangle to hexagon when the moisture ratios were about 0.78 and 0.36, while no shape change was detected in the case of agar cubes with 20% sucrose. Agar with 0% sucrose had almost no solids to support its structure after water had been evaporated. On the other hand, agar with 20% sucrose possessed more solids to support its structure and hence deformed more uniformly. In other words, the cubes with 20% sucrose experienced only the change in size; no convexity and concavity were observed.

**Table 2:** Top-view images of agar cubes with 0 and 20% sucrose during drying at 80 °C.

Moisture ratio	1.00 ± 0.00	0.78 ± 0.02	0.56 ± 0.03	0.36 ± 0.03	0.20 ± 0.02	0.06 ± 0.01
0% sucrose						
	Shape = Rectangle i = 100 j = 100 k = 100 l = 100 m = “Uniform” Shape_Ig_ = Rectangle Shape_CNN_ = Rectangle Extent = 0.9473 *D*_F_ = 2.1399	Shape = Rectangle i = 84.35 j = 96.84 k = 96.93 l = 101.11 m = “Non-uniform” Shape_Ig_ = Inclined rectangle Shape_CNN_ = Rectangle Extent = 0.9396 *D*_F_ = 2.1171	Shape = Rectangle i = 68.69 j = 84.98 k = 83.91 l = 102.76 m = “Non-uniform” Shape_Ig_ = Inclined rectangle Shape_CNN_ = Rectangle Extent = 0.9167 *D*_F_ = 2.0961	Shape = Hexagon i = 52.75 j = 77.47 k = 75.48 l = 103.32 m = “Non-uniform” Shape_Ig_ = Inclined rectangle Shape_CNN_ = Rectangle Extent = 0.8771 *D*_F_ = 2.0747	Shape = Pentagon i = 36.84 j = 65.22 k = 60.92 l = 102.15 m = “Non-uniform” Shape_Ig_ = Inclined rectangle Shape_CNN_ = Rectangle Extent = 0.8845 *D*_F_ = 2.0545	Shape = Pentagon i = 24.07 j = 58.01 k = 50.19 l = 99.33 m = “Non-uniform” Shape_Ig_ = Inclined rectangle Shape_CNN_ = Rectangle Extent = 0.7944 *D*_F_ = 2.0309
20% sucrose						
	Shape = Rectangle i = 100 j = 100 k = 100 l = 100 m = “Uniform” Shape_Ig_ = Inclined rectangle Shape_CNN_ = Rectangle Extent = 0.8637 *D*_F_ = 2.2786	Shape = Rectangle i = 79.27 j = 86.62 k = 84.81 l = 100.04 m = “Uniform” Shape_Ig_ = Inclined rectangle Shape_CNN_ = Rectangle Extent = 0.8613 *D*_F_ = 2.2423	Shape = Rectangle i = 69.24 j = 81.41 k = 81.27 l = 100.70 m = “Uniform” Shape_Ig_ = Inclined rectangle Shape_CNN_ = Rectangle Extent = 0.8627 *D*_F_ = 2.2508	Shape = Rectangle i = 62.40 j = 80.67 k = 82.33 l = 99.98 m = “Uniform” Shape_Ig_ = Inclined rectangle Shape_CNN_ = Rectangle Extent = 0.8712 *D*_F_ = 2.2615		

Shape_CNN_ was that obtained via CNN Xception.

Top-view shape changes of agar cubes with 0% sucrose could be divided into three periods. First, the shape of the cubes was slightly convexed. This is because their edges were hardened while the other portions, which were still moist and elastic, were forced to convexed out (see the results at the moisture ratios of 0.78 and 0.56). i decreased from 100 to 84.35 and 68.69% when the area decreased by 15.65 and 31.31%, while j decreased from 100 to 96.84 and 84.98%, indicating the horizontal contractions of 3.16 and 15.02%. k also decreased from 100 to 96.93 and 81.91%, indicating the vertical contractions of 3.07 and 18.09%. The decreasing rate of k was higher than that of j, implying that the vertical contraction was more extensive than the horizontal one. This is because the direction of drying air flow in the present experimental study was perpendicular to the top horizontal edges of the cubes. In the case of l, its value increased from 100 to 101.11 and 102.76%, indicating, as mentioned earlier, that convexity had occurred. The top-view shape of the cubes then appeared as rectangle rather than as square. Parameter m also correctly indicated the non-uniform deformation.

During the second period, when the moisture ratios were between 0.56 and 0.36, the shape of the cubes changed into hexagon. i decreased from 68.69 to 52.75%, indicating that shrinkage still occurred. The value of j decreased from 84.98 to 77.47%, while that of k decreased from 83.91 to 75.48%. Trends of changes of j and k were similar to those during the first period in that there was more extensive vertical contraction than the horizontal one. The value of l changed from 102.76 to 103.32%, indicating that there was also convexity. It was indeed this convexity that led to further shape change. Parameter m again indicated non-uniform deformation.

Finally, when the moisture ratios were lower than 0.36, structural collapse was noted, resulting in the change of shape of agar cubes from hexagon to pentagon. This is because agar cubes exhibited concavity in the middle softest region prior to finally being hardened (see the results at the moisture ratios of 0.20 and 0.06). The value of i decreased to 24.07%, implying that agar cubes extensively shrunk; j and k also decreased to 58.01 and 50.19%, respectively. The value of l decreased to 99.33%, implying that the cubes were concaved. Parameter m again indicated that the deformation was non-uniform in nature.

The results obtained by the developed algorithm were compared with those obtained using the algorithm developed by Igathinathane et al.^17^ and CNN Xception, in terms of Shape_Ig_ and Shape_CNN_, respectively; CNN Xception was selected due to its higher overall identification accuracy. The results obtained using Extent and fractal dimension were also compared. The results are as shown in Table 2. The developed algorithm could both identify and describe the changes of shape. The interpretation indeed agreed with that via visual observation. On the contrary, the algorithm of Igathinathane et al.^17^ could only identify the shape as “inclined rectangle” even though the cubes changed into other shapes (e.g., pentagon, hexagon) rather than rectangle. Interestingly, although CNN Xception could identify the shapes of computer-generated images very accurately, it could only identify the shape of agar cubes as “rectangle.” This might be due to the insufficient number of images for the network training. Although as many as 45,000 images were used for training the network, it still did not succeed in this real-world application. This illustrates a limitation of CNNs when being applied even to identify simple shapes of practical materials and/or objects. Acquiring a sufficient number of real-world images for training may not always be feasible, however.

Extent and fractal dimension could only identify the points where non-uniform deformation started to take place but lacked the ability to describe such a deformation. This agrees with the observation reported by Jinorose et al.^15^.

## Conclusion

A novel image analysis algorithm that can be used to identify shape and describe non-uniform deformation of an input image is proposed and was tested with both computer-generated images and cubes of agar gel, which was used as the test highly shrinkable material, undergoing drying. Identifying the shape of an input image without any alignment yielded the lowest accuracy. Among the tested alignment methods, the extrema method performed adequately when both the efficiency and required computational resource were taken into consideration. The algorithm was well capable of distinguishing the different deformation patterns of the computer-generated images and, in particular, agar cubes with different solids contents. In the latter case, the developed algorithm was superior to the use of simple shape parameters, shape-parameter based algorithm and even convolutional neural networks. It is important to note that the developed algorithm can identify any shape and its change without being limited to only predetermined shapes as in the case of other existing algorithms. The developed algorithm is robust and could be further developed for real-time process control of a drying process where size and shape of a material, in particular soft material, are of concern.

## Algorithm development and testing

### Algorithm development

#### Computer specification

Personal computer with Intel^®^ Core™ i7-10657G7 at 1.30 GHz was used in all cases. Computer is installed with NVIDIA GeForce GTX 1650 with Max-Q Design as Graphics Processing Unit with 32 GB of RAM and 512 GB of SSD. The operating system is Windows 10 Home.

#### Image pre-processing steps

Computer-generated images were first segmented using the algorithm described by Jinorose et al.^15^. First, artefact was eliminated from each image via the use of MATLAB^®^ (version R2020a, MathWork Inc., MA). Each image was then cropped to 512 × 512 pixel. Image segmentation was performed by converting an RGB image into a binary image using Otsu’s thresholding method^15^. Edge detection and holes filling were subsequently performed to extract an area of interest.

As in reality a material entering an image analysis system may initially be arbitrarily oriented, a material image must first be aligned into an axis prior to being analyzed. Three alignment methods were tested in this study viz. equivalent ellipsis, extrema and manual alignment methods (see Fig. 1). In the case of the equivalent ellipsis method, image would be rotated until α = 0°. Note that α is an angle between the major axis of an ellipse that has the same second moment of area of interest as that of an input image and the horizontal axis^31^ (Fig. 1a). In the case of the extrema method, image would be rotated until β = 0°. In this case, β is an angle between the line connecting points *p*_1_ and *p*_2_, which are the two extrema points, and the horizontal axis^28^ (Fig. 1b). In the case of the manual alignment method, image would be rotated from 0° to 360° at every 1° interval. The final angle of rotation (δ) was selected as the one that gave the lowest root mean square difference (RMSD) of the distance between the centroid and the edge of the image at angle θ (*d*_image,θ_) and that of the reference standard geometric shape (*d*_ref_,_θ_) at the same angle θ (Eq. 1).

1 $$\mathrm{RMSD}= \sqrt{\frac{\sum_{\uptheta =0}^{360}{({d}_{\mathrm{ref},\uptheta }-{d}_{\mathrm{image},\uptheta })}^{2}}{360}}$$

The final angle of rotation was then used to rotate the image as per the following rotation matrix (Eq. 2) using the ‘affine2d’ and ‘imwarp’ functions in MATLAB R2020a^31^:

2 $$\text{Rotation matrix}=\left[\begin{array}{ccc}\mathrm{cos}& -\mathrm{sin}& 0\\ \mathrm{sin}& \mathrm{cos}& 0\\ 0& 0& 1\end{array}\right]$$

#### Matching input image with reference geometric shapes

**Figure 4:**
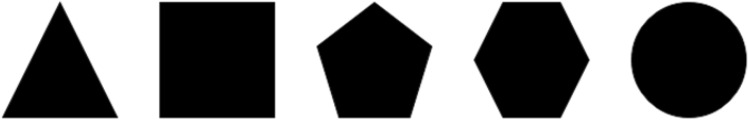
Reference geometric shapes used in this study.

Five reference geometric shapes were used in this study, i.e., triangle, rectangle, pentagon, hexagon and circle (Fig. 4). Each image itself was with the dimensions of 97 × 97 pixel, while the black background where the image sat on was with the dimensions of 512 × 512 pixel. An input image was matched to one of these geometric shapes to identify the closest shape of the image.

To identify the shape of an input image, its bounding box would be expanded until the length and width of that bounding box equal to those of the box belonging to all the reference geometric shapes. RMSD was then calculated and the most appropriate shape was selected as the one giving the lowest RMSD value.

#### Description of non-uniform deformation of input image

Before being able to describe non-uniform deformation of an input image, reference geometric shapes were first made to deform in various ways. The input image was first compared with the one with no deformation. The deformation patterns include swelling and shrinkage, horizontal and vertical elongations/contractions as well as convexity and concavity.

Swelling and shrinkage (or negative swelling) were achieved as per the swelling matrix (Eq. 3)^14^. Elongation, both in horizontal and vertical directions, was achieved as per the elongation matrices (Eqs. 4 and 5)^14^, while convexity and concavity functions are defined as shown in Eqs. (6) and (7)^32^. Built-in ‘affine2d’ and ‘geometricTransform2d’ functions were used along with the above-mentioned equations to achieve the deformation; ‘imwarp’ function was used to implement all the deformation operations.

3 $$\mathrm{Swelling \,matrix}=\left[\begin{array}{ccc}S& 0& 0\\ 0& S& 0\\ 0& 0& 1\end{array}\right]$$

4 $$\mathrm{Horizontal \,elongation \,matrix}=\left[\begin{array}{ccc}S& 0& 0\\ 0& 1& 0\\ 0& 0& 1\end{array}\right]$$

5 $$\mathrm{Vertical \,elongation \,matrix}=\left[\begin{array}{ccc}1& 0& 0\\ 0& S& 0\\ 0& 0& 1\end{array}\right]$$

6 $${d}_{\mathrm{convex}}={d}_{\mathrm{image},\uptheta }+ {d}_{\mathrm{image},\uptheta }^{3}\left(\frac{S}{{\mathrm{max}({d}_{\mathrm{image},\uptheta })}^{2}}\right)$$

7 $${d}_{\mathrm{concave}}={d}_{\mathrm{image},\uptheta }- {d}_{\mathrm{image},\uptheta }^{3}\left(\frac{S}{{\mathrm{max}({d}_{\mathrm{image},\uptheta })}^{2}}\right)$$

where *S* is the scaling factor.

All the deformation patterns are summarized in Table 3. Parameters i, j, k, l and m are used to describe the various deformation patterns. Parameter i (Eq. 8) indicates swelling and shrinkage; ‘+’ indicates swelling, while ‘−’ indicates shrinkage. Parameter j (Eq. 9) indicates horizontal elongation (+) and contraction (–). Parameter k (Eq. 10) indicates vertical elongation (+) and contraction (–). Parameter l (Eq. 11) indicates convexity (+) and concavity (–). Finally, parameter m is used to indicate whether the deformation is uniform or not. This latter parameter was calculated from Eq. (13) and the aforementioned parameter l; when α is less than 0.05 and l is less than 0.01, m would indicate uniform deformation. The choices of these decision criteria is as explained in the “Results and discussion” section.

**Table 3:** Deformation patterns of reference geometric shapes.

Deformation pattern	+	0	−
Swelling and shrinkage			
i, j, k, l, m system	+, +, +, 0, 0	0, 0, 0, 0, 0	−, −, −, 0, 0
Horizontal elongation and contraction			
i, j, k, l, m system	+, +, 0, 0, 1	0, 0, 0, 0, 0	−, −, 0, 0, 1
Vertical elongation and contraction			
i, j, k, l, m system	+, 0, +, 0, 1	0, 0, 0, 0, 0	−, 0, −, 0, 1
Convexity and concavity			
i, j, k, l, m system	0, 0, 0, +, 1	0, 0, 0, 0, 0	0, 0, 0, −, 1

8 $$\mathrm{i}= \frac{A-{A}_{\mathrm{ini}}}{{A}_{\mathrm{ini}}}$$

9 $$\mathrm{j}= \frac{L-{L}_{\mathrm{ini}}}{{L}_{\mathrm{ini}}}$$

10 $$\mathrm{k}= \frac{W-{W}_{\mathrm{ini}}}{{W}_{\mathrm{ini}}}$$

11 $$\mathrm{l}= \frac{{\mathrm{Extent}}_{\mathrm{Ellipse}}- {\mathrm{Extent}}_{\mathrm{Ellipse},\mathrm{ini}}}{{\mathrm{Extent}}_{\mathrm{Ellipse},\mathrm{ini}}}$$

12 $${\mathrm{Extent}}_{\mathrm{Ellipse}}= \frac{A}{\frac{\pi }{4}(\mathrm{major})(\mathrm{minor})}$$

13 $$\mathrm{\upalpha }= \frac{{\mathrm {j-k}}}{\mathrm{min}{\mathrm (j,k)}}\times 100$$

In the above equations, *A* is the projected area of the area of interest (AOI), *L* is the length of the bounding box of AOI, *W* is the width of the bounding box of AOI. ‘major’ and ‘minor’ are the lengths (in pixel) of the major axis and minor axis, respectively, of the ellipse that has the same normalized second central moment as that of AOI.

#### Comparison between developed and existing algorithms

Performance of the developed algorithm was compared with that of algorithm developed by Igathinathane et al.^17^. These investigators developed the algorithm that could identify an image into either triangle, rectangle or circle. Three shape parameters, i.e., reciprocal aspect ratio (RAR), rectangularity as well as Feret major ratio (FMR), which were calculated as per Eqs. (14–16), were used for such an identification purpose. Each of these parameters was assigned a specific range over which each of the three shapes (Shape_Ig_) would be identified. Note that if the shape of an input image was far from the above three standard shapes, the algorithm would fail to capture such a shape; all peculiar shapes would be identified as an ‘inclined rectangle.’

14 $$\mathrm{RAR}= \frac{\mathrm{minor}}{\mathrm{major}}$$

15 $$\mathrm{Rectangularity}= \frac{\frac{\pi }{4}(\rm major)(\rm minor)}{{A}_{\mathrm{BB}}}$$

16 $$\mathrm{FMR}= \frac{{D}_{\mathrm{f}}}{\mathrm{major}}$$

where *A*_BB_ is the area of a rectangular bounding box, while *D*_f_ is the maximum Feret diameter.

For non-uniform deformation description, Extent (Eq. 17)^15,31^ and fractal dimension (Eq. 18)^33^ were also used. The description results based on these parameters were compared with those given by the newly developed algorithm.

17 $$\mathrm{Extent}= \frac{A}{{A}_{\mathrm{BB}}}$$

18 $$\mathrm{Fractal\, dimension }({D}_{\mathrm{F}})= \frac{\mathrm{log}{(N}_{r})}{\mathrm{log}\left(\frac{1}{r}\right)}$$

where *N*_*r*_ is the number of boxes intercepted with the size of each iteration of *r* value, while *r* is the size of the box.

Finally, convolutional neural networks (CNNs) with two different architectures, i.e., ResNet-50 and Xception, were tested via the use of the Deep Learning toolbox in MATLAB^®^ (version R2020a, MathWork Inc., MA). Stochastic gradient descent with momentum (SGDM) with InitialLearnRate of 0.01 were used^34^. The validation frequency was 315, while the MaxEpochs and MinBatchSize were both set at 10.

In the cases of the presently developed algorithm and that of Igathinathane et al.^17^ 4200 computer-generated images were created and used to test the algorithms. On the other hand, in the case of CNNs, 45,000 computer-generated images were created and used to train (31,500 images) and validate (13,500 images) the networks. The same 4200 images were also used to test the trained CNNs.

### Agar drying

#### Agar preparation

2% (w/w) granulated purified agar (Product no. 1016141000, Merck Millipore Corp., Darmstadt, Germany) was mixed with either 0 or 20% (w/w) sucrose solution. The mixture was stirred at room temperature (25 ± 2 °C) at 100 rpm for 1 h, then heated to 95 °C and finally stirred at 150 rpm for 10 min. The mixture was cooled at room temperature until its temperature reached 50 °C. The cooled mixture was poured into a silicone mold to form agar cubes, each with the dimensions of 1.9 × 1.9 × 1.9 cm. The sample was allowed to set at room temperature for 1 h before being kept at 4 ± 1 °C for 24 h.

#### Drying experiments

Drying experiments were conducted in a hot air dryer (Memmert GmbH+ Co. KG, UM500, Schwabach, Germany) at 80 °C. Three agar cubes were taken out for moisture content determination and image acquisition at every 1 h.

#### Moisture content determination

A sample was weighed using a digital balance with an accuracy of ± 0.0001 g (Sartorius Lab Instruments GmbH & Co. KG, BSA224S-CW, Göttingen, Germany) and then dried in a hot air oven (Memmert GmbH+ Co. KG, UM500, Schwabach, Germany) until constant mass was obtained as per AOAC method 984.25 (2000). The moisture content (MC) of the sample was calculated using Eq. (19):

19 $$\mathrm{MC }\left(\mathrm{\% d}.\mathrm{b}.\right)= \frac{{m}_{\mathrm{i}}-{m}_{\mathrm{bd}}}{{m}_{\mathrm{bd}}}\times 100\%$$

where *m*_i_ is the mass of agar before drying at 105 °C, while *m*_bd_ is the bone-dry mass.

#### Image acquisition and segmentation

The utilized image acquisition system was the one described by Jinorose et al.^15^. Only top-view images were acquired and analyzed. Image segmentation was implemented as also described by Jinorose et al.^15^.

## Data Availability

Experimental and relevant data are available from the authors upon suitable request.
